# Urinary mycoestrogens and age and height at menarche in New Jersey girls

**DOI:** 10.1186/s12940-019-0464-8

**Published:** 2019-03-22

**Authors:** Zorimar Rivera-Núñez, Emily S. Barrett, Elizabeth A. Szamreta, Sue A. Shapses, Bo Qin, Yong Lin, Helmut Zarbl, Brian Buckley, Elisa V. Bandera

**Affiliations:** 1 Rutgers Cancer Institute of New Jersey, 195 Little Albany St., New Brunswick, NJ 08901 USA; 2 Environmental and Occupational Health Sciences Institute (EOHSI), Rutgers University, 170 Frelinghuysen Rd, Piscataway, NJ 08854 USA; 3 Rutgers School of Public Health, 683 Hoes Lane West, Piscataway, NJ 08854 USA; 4 Department of Nutritional Sciences, Rutgers University, 65 Dudley Rd, New Brunswick, NJ 08901-8520 USA

**Keywords:** Zearalenone, (α-ZAL), Mycoestrogens, Puberty, Endocrine disruptors

## Abstract

**Background:**

Despite evidence of the endocrine disrupting properties of zearalenone (ZEN) and alpha-zearalanol (zeranol, α-ZAL), they have been minimally studied in human populations. In previous cross-sectional analyses, we demonstrated that 9–10 years old girls with detectable urinary ZEN were of shorter stature and less likely to have reached the onset of breast development than girls with undetectable urinary ZEN. The aim of this study was to examine baseline concentrations of ZEN, (α-ZAL), and their phase-1 metabolites in relation to subsequent growth and timing of menarche using 10 years of longitudinal data.

**Methods:**

Urine samples were collected from participants in the Jersey Girl Study at age 9–10 (*n* = 163). Unconjugated ZEN, (α-ZAL), and their metabolites were analyzed using high performance liquid chromatography and tandem mass spectrometry. Information on height, weight, and pubertal development was collected at a baseline visit with annual follow-up by mail thereafter. Cox regression was used to evaluate time to menarche in relation to baseline ZEN, (α-ZAL), and total mycoestrogen exposure. Z-scores for height and weight were used in mixed models to assess growth.

**Results:**

Mycoestrogens were detectable in urine in 78.5% of the girls (median ZEN: 1.02 ng/ml, range 0–22.3). Girls with detectable urinary concentrations of (α-ZAL) and total mycoestrogens (sum of ZEN, (α-ZAL) and their metabolites) at baseline were significantly shorter at menarche than girls with levels below detection (*p* = 0.04). ZEN and total mycoestrogen concentrations were inversely associated with height- and weight-z-scores at menarche (adjusted *β* = − 0.18, 95% CI: -0.29, − 0.08, and adjusted *β* = − 0.10, 95% CI: -0.21, 0.01, respectively).

**Conclusion:**

This study supports and extends our previous results suggesting that exposure to ZEN, (α-ZAL), and their metabolites is associated with slower growth and pubertal development in adolescent girls.

## Introduction

Puberty is a critical developmental window during which there are rapid and significant changes in maturation and activation of multiple hormone axes [1, 2]. These changes make puberty a particularly susceptible time period for endocrine disruption. Exposures during this critical window can impact the physiology of female reproductive organs and the hypothalamic-pituitary-ovarian hormonal axis, including abnormal pubertal changes and ultimately, increased susceptibility to hormone-sensitive cancers including breast cancer [3].

Zearalenone (ZEN) is a mycotoxin produced by certain fungal species of the genus *Fusarium,* while alpha-zearalanol (zeranol, α-ZAL) is a synthetic derivative of ZEN as well as a product of ZEN metabolism. ZEN and α-ZAL are widely found in the food supply, where exposure may be deliberate (e.g. α-ZAL administered to livestock as a growth promoter) or inadvertent (e.g. fungal contamination of grain products by ZEN). Consequently, both ZEN and α-ZAL can be found through a range of food products including cereal, meat, milk, wine, beer, dried fruit and spices. Although these mycoestrogens have short half- lives (< 24 h) [4], continuous exposure through the diet presents a public health concern. In livestock, ZEN, α-ZAL, and their metabolites (i.e., α-zearalenol, β-zearalenol, zearalanol, β-zearalanol) are generally present due to the consumption of contaminated feed. These mycoestrogens have demonstrated endocrine disrupting effects in livestock and exposure can disrupt estrous cycling, resulting in decreased fertility [5, 6]. In fact, ZEN and zearanol are recognized as phytoestrogens, naturally occurring compounds that mimic the activity of estrogen, causing changes in hormone production and balance [7–9]. Despite widespread exposure to these compounds through diet [10–12], little is known about the effects of ZEN and α-ZAL exposures in human populations, particularly during critical windows of exposure such as puberty.

Several small studies, primarily in clinical populations, have measured ZEN exposure during the prepubertal period [13–15]. An Italian study (*n* = 63) examined 32 girls with central precocious puberty and 31 healthy controls [13]. Girls with detectable levels of ZEN (*n* = 6) and α-zearalenol were taller and proportionally heavier (*p* < 0.01) than girls with undetectable levels of these mycoestrogens. Asci et al. measured ZEN levels in girls living in the Mediterranean area of Turkey (*n* = 78) [14]. Girls were divided into three groups: girls with premature thelarche (PT), girls with idiopathic precocious puberty (IPP), and a control group. The authors reported that girls in the PT group had twice the levels of ZEN compared to the control group (*p* = 0.07). Similarly, girls in the IPP group had 2.8 times the levels of ZEN compared to the control group (*p* < 0.05). In the Jersey Girl study, a cross-sectional study of healthy 9–10 year old girls in the U.S., we previously reported that peripubertal girls with detectable ZEN in urine were of shorter stature (*p* < 0.0001) and less likely to have reached the onset of breast development than girls with undetectable ZEN (Prevalence ratio: 0.79, 95% confidence interval [95% CI]: 0.60, 1.04) [16]. We now extend these findings in the Jersey Girl Study cohort using follow-up data gathered for up to 10 years post-baseline. The aim of the current analysis is to examine baseline levels of ZEN, α-ZAL, and their phase-1 metabolites in relation to subsequent growth and onset of menarche. To our knowledge, this is the first longitudinal analysis of urinary mycoestrogens and pubertal outcomes in healthy girls.

## Methods

### Population

Study procedures for the Jersey Girl Study have been described in detail elsewhere [16]. In brief, 202 girls were recruited through pediatric practices, flyers, and word of mouth throughout the state of New Jersey between 2006 and 2014. Eligibility criteria included: female, age 9–10 years, English-speaking, in good health, no cognitive impairments, and living with biological mother. Parental consent and girl’s assent were obtained before data collection began. The study was approved by the Rutgers Health Sciences Institutional Review Board. Analyses in the current study included 163 girls with mycoestrogens measures.

### Baseline questionnaire & visit

Interested mothers completed a brief questionnaire over the phone to determine study eligibility. Once mothers and daughters were consented to the study, they completed a clinic visit during which trained staff collected data on anthropometry and pubertal development. Girls brought in first morning void urine samples to their visit. A self-administered baseline questionnaire was completed by the girls’ mothers, with questions on demographic factors, environmental exposures, medical history, and prenatal and early childhood factors.

### Follow-up questionnaires

Annual follow-up questionnaires were mailed to participants until the onset of menses. On each questionnaire, girls and/or mother reported girls’ current height, weight, and date at menarche (if applicable). The number of follow-up questionnaires completed ranged from 2 to 7 with most girls (83%) had 4–5 measurements within the follow-up period. This analysis includes follow-up data through December 2017. For 25 girls age at menarche was unknown, either because they have not reached menses yet (*n* = 9) or they have been lost to follow-up (*n* = 16).

### Exposure data

Uunconjugated urinary ZEN, α-ZAL, and their metabolites were measured at baseline in urine samples from the first 163 participants enrolled in the study. Participants were instructed to collect a first morning void urine sample (using provided collection cups) and to bring it to the in-person baseline study visit. Samples were aliquoted and stored at − 70 °C, until analysis at the Chemical Analysis Facility Core of the Environmental and Occupational Health Sciences Institute (EOHSI) at Rutgers University. The analytical protocol has been previously described [16]). Briefly, free ZEN, α-ZAL and their metabolites were measured using High Performance Liquid Chromatography (HPLC) and tandem mass spectrometry (MS/MS) A Thermo Surveyor HPLC with a Hypersil C18 column were coupled to a Thermo LTQ linear ion trap mass spectrometer were used for the analysis. Commercial standards were used to define the calibration curve. The limit of detection (LOD) was 0.05 ng/ml. Concentrations of all analytes were corrected for urine dilution by specific gravity [17].

Our primary models considered ZEN and zearanol individually. Secondly, we summed all of the analytes [aglycones] (ZEN, zearanol, and their related metabolites, i.e., α-zearalenol, β-zearalenol, β-zearalanol, and zearalanol) into a composite measure (“total mycoestrogens”). We considered concentrations continuously and also categorized participants by status (ZEN, α-ZAL, and total mycoestrogen above LOD, versus below LOD). Values below LOD were assigned a value of the LOD divided by the squared root of two [18].

### Statistical analysis

We examined univariate statistics (means, medians, percentages, interquartile ranges (IQR) for all variables of interest. We compared mycoestrogen concentrations in relation to participant characteristics using Kruskal-Wallis tests. Mycoestrogen concentrations were log transformed to approximate normality for all statistical models. Two sets of models were fit examining 1) age at menarche/time to menarche; and 2) height and weight at menarche. For the menarche outcome, time to menarche was calculated as the duration between the baseline visit and the date of menarche as reported on the annual follow-up questionnaires. Loss to follow-up girls and girls with no menarche yet were right censored. In our primary models, unadjusted and adjusted Cox regression models were used to estimate hazard ratios (HRs) and 95% CIs for time to menarche in relation to mycoestrogen concentrations at baseline. Secondarily, we examined early menarche, defined as onset of menses at or before 11.8 years of age (corresponding to the 25th percentile of the age at menarche distribution of all girls reporting age at menarche). To look at early menarche, we fit unadjusted and adjusted logistic regression to estimate odds ratios (ORs) and 95% CIs. Thirty-four girls met the early menarche definition and a total of 139 girls with complete menarche information were included in this analysis. For Cox and logistic regression models, exposure was dichotomized into detectable and undetectable mycoestrogen concentrations at baseline. Mixed effect linear models with an unstructured correlation matrix were used to assess average height- and weight-z-scores (continuous) in relation to mycoestrogen levels. These models included repeated measures for height- and weight-z-scores, consequently models calculate estimates using longitudinal height- and weight-z-scores for each girl.

The following potential confounders were considered in all multivariable models: girl’s age at recruitment, race, maternal education, family income, county of residence, mid-parental height, and body mass index (BMI) at menarche. Mid-parental height was calculated as [(mother’s height now + father’s height now) + 2.5]/2 [19]. In models examining menarcheal outcomes, we additionally considered BMI at menarche as a potential covariate. BMI was calculated as weight (kg) over height (m) squared (at the follow-up time where menarche was reported) and we used CDC sex- and age-specific tables to categorize subjects as: underweight (BMI <5th percentile), normal weight (BMI ≥5th - < 85th percentile), overweight (BMI ≥ 85th - ≤95th percentile), and obese (BMI > 95% percentile). Confounding factors were separately evaluated based on percentage change in regression coefficient estimates in each multivariable model (ZEN, α-ZAL, total mycoestrogens) compared to bivariate models, and only those variables changing the coefficient estimate > 10% were retained in final models.

Survival and logistic regression models examining menarcheal outcomes were adjusted for family income and BMI at menarche. Because BMI could potentially be in the causal pathway in time to menarche analyses, it may not be appropriate to adjust for it. Therefore, two adjusted Cox regression models are presented, 1) Model A adjusted for only family income, and 2) Model B adjusted for family income as well as BMI at menarche. Mixed effect linear models examining height- and weight- z-scores as main outcomes were adjusted for mid-parental height and family income. All statistical analyses used SAS 9.4 (SAS Institute, Cary, NC, USA). Statistical significance was based on alpha < 0.05.

## Results

ZEN was detected in 55% of the samples, α-ZAL in 20% for and total mycoestrogens in 78%. [16]. Corrected arithmetic mean values and standard deviations were as follows: ZEN: 0.57 (0.07) ng/ml; α-ZAL: 0.21 (0.03) ng/ml; α-zearalenol: 0.13(0.02) ng/ml; β-zearalenol: 0.29 (0.03) ng/ml; β-zearalanol: 0.24 (0.04) ng/ml; zearalanone: 0.21 (0.03) ng/ml, total mycoestrogens: 0.89 (0.06) ng/ml. Table 1 shows medians and IQRs for ZEN, α-ZAL, and total mycoestrogens only for demographic variables relevant to the current study and new outcome variables. Girls with higher urinary concentrations of ZEN (*p* = 0.04), α-ZAL (*p* = 0.04), and total mycoestrogens (*p* = 0.07) were shorter at menarche than girls with lower concentrations. We compared characteristics of girls lost to follow-up with those with complete follow-up data. Girls lost to follow up were similar with respect to age at baseline, race, and baseline BMI than those with complete data. Mothers’ of girls with complete data were slightly more likely to have a graduate degree.

**Table 1:** Medians and interquartile ranges for unconjugated ZEN, zeranol and total mycoestrogens according to selected characteristic among the Jersey Girl Study participants included in these analyses (*n* = 163)

Characteristic	n (%)	ZEN Median(IQR) (ng/ml)	Zeranol Median(IQR) (ng/ml)	Total Mycoestrogen Median(IQR)(ng/ml)
Girl’s Race				
White	146 (90)	0.15 (0.35)	0.04 (0)	0.30 (0.71)
non-White	13 (8)	0.31 (0.66)	0.10 (0.20)	0.53 (1.66)
Missing	4 (2)	1.10 (1.28)	0.04 (0.17)	1.25 (1.74)
*p*-value		0.47	0.23	0.26
BMI at Menarche for Age and Gender (CDC)^a^				
Underweight	9 (6)	0.04 (0)	0.04 (0)	0.30
Healthy Weight	101 (62)	0.23 (0.28)	0.04 (0)	0.24 (0.51)
Overweight	16 (10)	0.10 (0.36)	0.04 (0)	0.26 (0.66)
Obese	12 (7)	0.30 (0.65)	0.04 (0)	0.15 (0.41)
Missing	25 (15)	0.23 (0.41)	0.04 (0)	0.30 (0.39)
*p*-value		0.49	0.52	0.77
Age at Menarche (years)				
≤ 11.8^b^	59 (22)	0.22 (0.43)	0.04 (0)	0.31 (0.70)
> 11.8	104 (63)	0.14 (0.32)	0.04 (0)	0.32 (0.89)
Missing	25 (15)	0.24 (0.39)	0.04 (0)	0.37 (0.67)
*p*-value		0.49	0.53	0.33
Weight at Menarche (Kg)^c^				
≤ 46.5	46 (28)	0.22 (0.49)	0.04 (0.05)	0.30 (0.86)
> 46.5–52.6	46 (28)	0.09 (0.30)	0.04 (0)	0.31 (0.59)
> 52.6	46 (28)	0.09 (0.54)	0.04 (0)	0.26 (0.87)
Missing	25 (15)	0.22 (0.39)	0.04 (0)	0.40 (0.83)
*p*-value		0.64	0.53	0.27
Height at Menarche (cm)^c^				
≤ 156.2	49 (30)	0.27 (0.56)	0.04 (0.07)	0.49 (0.90)
> 156.2–162.6	52 (32)	0.17 (0.36)	0.04 (0)	0.28 (0.78)
> 162.6	37 (23)	0.04 (0.29)	0.04 (0)	0.12 (0.44)
Missing	25 (15)	0.22 (0.39	0.04 (0)	0.40 (0.83)
*p*-value		0.04	0.04	0.07

Notes: *p* values from Kruskal-Wallis tests, levels below the LOD were replaced by [LOD/√2] (medians = 0.04 are <LOD), and missing *n* included girls lost to follow-up and those with no menses yet

Total Mycoestrogen [aglycones]: sum of zearalenone, zeranol, α-zearalenol, β-zearalenol, β-zearalanol, and zearalanol, *ZEN* Zearalenone, *IQR* interquartile range

^a^Underweight (<5th Percentile), Healthy Weight (5th - <85th percentile), Overweight (85th- < 95th percentile), Obese (>95th percentile) percentile)

^b^25th percentile of age at menarche distribution among all girls with recorded age at menarche

^c^Categories are based on tertiles for weight and height distribution at the follow-up date when menarche was reported

Average age at menarche was 12.8 years and 34 girls met the early menarche definition. Although not statistically significant, results of unadjusted models suggested that girls with detectable urinary α-ZAL tended to have longer time to menarche compared to girls with undetectable levels (Table 2). In adjusted models, ZEN and total mycoestrogen results were largely null suggesting no effect by BMI. The results of our secondary logistic regression analyses examining early menarche were similar and indicated no relationship between baseline >LOD mycoestrogen concentrations and early menarche (not shown) and led to similar conclusions compared to our primary Cox regression analysis.

**Table 2:** Cox regression models for the association between mycoestrogen exposure (ng/ml) and time to menarche in the Jersey Girl Study

Mycoestrogen (ng/ml)	n	Unadjusted HR (95%CI)	Adjusted^a^ HR(95%CI)	Adjusted^b^ HR(95%CI)
ZEN				
< LOD	62	Ref	Ref	Ref
> LOD	77	1.20 (0.70–2.08)	1.11 (0.78–2.0)	0.99 (0.93–1.05)
Zeranol				
< LOD	108	Ref	Ref	Ref
> LOD	31	0.35 (0.06–1.89)	0.41 (0.10–1.85)	0.35 (0.06–2.00)
Total Mycoestrogen				
< LOD	31	Ref	Ref	Ref
> LOD	108	1.00 (0.96–1.04)	0.94 (0.89–1.00)	0.99 (0.95–1.04)

*HR* Hazard Ratio, *CI* Confidence Interval, *LOD* limit of detection (0.05 ng/ml), REF: reference group, Total Mycoestrogen [aglycones]: sum of zearalenone, zeranol, α-zearalenol, β-zearalenol, β-zearalanol, and zearalanol, ZEN: Zearalenone

^a^Adjusted for family income

^b^Adjusted for family income and body mass index at menarche

Table 3 shows β and 95% CIs from mixed effect linear models. All mycoestrogen levels at baseline showed negative associations with average weight- and height-z-scores during the time between enrollment and menarche (unadjusted and adjusted models [for mid-parental height and family income]). α-ZAL levels were significantly associated with lower average height (adjusted *β* = − 0.23, 95%CI: -0.37,-0.08) and weight (adjusted *β* = − 0.18, 95%CI: -0.29,-0.08) at menarche z-scores in adjusted models. Similarly, total mycoestrogen levels were significantly associated with lower height (adjusted *β* = − 0.17, 95%CI: -0.33,-0.09) at menarche z-scores but not with weight at menarche z-scores. None of the examined associations were significant for ZEN concentrations considered individually.

**Table 3:** Mixed effect linear models for the association between mycoestrogen exposure (ng/ml) and height and weight z-scores at menarche in the Jersey Girl Study (*n* = 163)

	Height z-score		Weight z-score	
	Unadjusted β (95%CI)	Adjusted ^a^ β (95%CI)	Unadjusted β (95% CI)	Adjusted ^a^ β (95% CI)
Mycoestrogen				
ZEN	−0.03 (−0.07,0.01)	−0.02 (−0.08,0.03)	−0.02 (−0.10,0.05)	−0.02 (−0.11,0.07)
Zeranol	−0.25 (−0.37,-0.12)	−0.23 (− 0.37,-0.08)	− 0.16 (− 0.28,-0.05)	−0.18 (− 0.29,-0.08)
Total Mycoestrogen	− 0.19 (− 0.31,-0.07)	−0.17 (− 0.33,-0.09)	−0.10 (− 0.21,-0.05)	−0.09 (− 0.21,0.01)

*CI* Confidence Interval, Total Mycoestrogen [aglycones]: sum of zearalenone, zeranol, α-zearalenol, β-zearalenol, β-zearalanol, and zearalanol, ZEN: Zearalenone

^a^Model adjusted for mid-parental height and family income

## Discussion

This study is the first longitudinal study to evaluate ZEN exposure in relation to age at menarche and growth in healthy peri-pubertal girls. We observed that girls with detectable mycoestrogen levels were significantly shorter in stature at menarche than girls with mycoestrogen levels below the LOD. Girls with detectable α-ZAL concentrations were also less likely to have early menarche, but results were not statistically significant. The current study has expanded the findings from our previous report suggesting delayed pubertal development (i.e., thelarche) in girls with detectable mycoestrogen levels compared to girls with levels below the LOD [16].

Very few studies have measured mycoestrogen levels in relation to pubertal timing or growth [13–16]. Beyond our previous work in this population, a single study has evaluated body size [13] and no study has evaluated age at menarche. Our results contrast with those reported by Massart et al. suggesting that ZEN and α-zearalenol levels were positively associated with height and weight in girls. However, they measured mycotoxins in serum from girls (6–7 years old) participating in an idiopathic central precocious puberty case-control study. Additionally, they had a smaller sample size (*n* = 63) and only 6 girls had detectable levels of ZEN and α-ZAL. Finally, for those 6 girls with detectable serum ZEN levels, exposure (median ZEN: 0.12 ng/ml) was 10-fold lower than our measures in urine (median ZEN: 1.02 ng/ml). Thus given the differences in population, age at assessment, biospecimen type, and exposure levels, results of the two studies may not be readily comparable.

A larger literature has examined peri-pubertal growth and menarche in relation to other endocrine disruptors that act on sex steroid pathways. For example, in one study of adolescent girls, high intake of isoflavones, the predominant dietary phytoestrogens, was associated with older age at breast development [20]. Similarly in a diverse urban cohort, girls with higher levels of urinary phytoestrogens (i.e., daidzein, genistein, enterolactone) were less likely to have experienced Tanner Breast Stage 2+ than girls with low levels [21, 22]. Most recently, in the same cohort, Wolff et al. examined age at menarche in relation to urinary concentrations of phthalates and phenols, two classes of chemicals linked to altered estrogenic activity [23]. They showed that 2,5-Dichlorophenol was associated with early menarche (HR: 1.34; 95% CI: 1.06, 1.71) whereas enterolactone (HR: 0.82; 95% CI: 0.66, 1.03) and mono-3-carboxypropyl phthalate (HR: 0.73: 95% CI: 0.59, 0.91) were associated with later menarche. Finally, higher perfluorooctanoic acid (PFOA) and perfluorooctane sulfonate (PFOS) levels have been associated with delayed age of menarche [24]. Our results suggest that ZEN may have anti-estrogenic properties similar to those demonstrated in the phytoestrogen studies [20, 21, 23]. Like phytoestrogens, ZEN may inhibit the activity of key enzymes in the steroidogenic pathway including aromatase [25] and 17β-hydroxysteroid dehydrogenase [26], but more research is needed to fully understand these pathways.

Human exposure to ZEN is predominantly through food sources. ZEN can be found in the entire food chain through different products including cereal, milk, meat, dried fruit and spices. Although ZEN is not regulated in the U.S., the European Food Safety Authority (EFSA) established the tolerable daily intake for ZEN as 0.25 μg/kg body weight per day [12]. The European Commission also regulates limits for human consumption products (e.g., maize, cereals) and animal feed (e.g., maize, cereal, complementary feeding stuff). Our previous report suggested that beef and popcorn are the main sources of ZEN exposure in our study population [16], and meat (all types) was the primary source of ZEN exposure in local adult women [27], but additional work is needed to confirm these results and identify additional common sources of dietary exposure in humans.

ZEN metabolism plays a significant role in ZEN toxicity. ZEN follows two major biotransformation pathways: hydroxylation and conjugation [28]. Hydroxylation leads to α-zearalenol and β- zearalenol as well as α-ZAL, β-zearalanol, and zearalanone. Some of these hydroxylation metabolites tend to be more toxic than ZEN [29]. For example, in reporter gene assays examining estrogenic potency, ZEN was ~ 70 times less potent than α –zearalenol but twice as potent as β–zearalenol [29]. Conjugation results in ZEN glucuronide conjugates that are less toxic than ZEN. An in vitro study assessing ZEN biotransformation, reported that formation of ZEN glucuronides is a detoxification pathway that may attenuate estrogenic effects [30]. The proportion of ingested ZEN that goes through detoxification is not well understood and the relative contributions of the two biotransformation pathways may have different effects in human health. In the current study, ZEN urinary concentrations were higher than those of α-ZAL and other metabolites, suggesting that a portion of ZEN is excreted with no biotransformation. Other biomonitoring studies in adults have reported that some of the metabolites levels in urine are comparable or exceeded those of ZEN [31, 32]. We were not able to measure conjugated ZEN (or metabolites) which may explain the higher number of participant with undetectable levels of α-ZAL and other metabolites. Additionally, it may increase the potential for measurement error in our estimates. Exposure misclassification may be larger in the Cox regression results where exposure was categorized (<LOD, >LOD). It is difficult to speculate the effect of exposure misclassification in our results given the very limited toxicokinetics data on ZEN in humans. Furthermore, human biotransformation of these compounds may be influenced by sex, age, and diet adding to the variability of the biomarker. Additional biomarker(s) studies are needed to further investigate ZEN metabolism and toxicity.

It is well known that exposures during early life including childhood and puberty can influence cancer risk [1, 33]. For example, an estimated 10% reduction in breast cancer risk is expected for each two-year increase in age at menarche [34]. Growth metrics (including height and height velocity) during childhood have also been associated with cancer risk including breast, endometrial, testicular and prostate cancers [35–38]. Pubertal growth is mostly stimulated by estradiol and experimental studies have shown that estrogen may affect epiphyseal maturation, normal skeleton proportions, and bone mineralization [39–41]. Furthermore, there is some evidence that at low concentrations estrogens may accelerate linear growth whereas at high levels may stop linear growth [40]. ZEN is a structural estrogen analogue, able to mimic the activity of naturally occurring estrogens [9]. It has been hypothesized that ZEN can exert its estrogenic effect by affecting pituitary and gonadal function [9]. Experimental studies have shown that ZEN affects breast cancer development and progression through its estrogenicity [42–44]. In contrast with some of the experimental evidence, our results suggest that ZEN may also have anti-estrogenic properties. Differences in exposure levels (which are typically much higher in animal models than we observed in our study) and timing of exposure may also be important to consider. It is possible, for instance, that exposure to ZEN and/or its metabolites during critical developmental periods may suppress the production of 17β-hydroxysteroid dehydrogenase, ultimately resulting in anti-estrogenic properties.

Our study has several strengths and limitations of note. This is the first longitudinal study to evaluate the relationship between ZEN exposure and growth and pubertal outcomes in girls and we were able to retain 85% of participants through menarche. At the same time, like the other existing epidemiological studies of mycoestrogens, we had a relatively small sample size and exposure was based on a single urine sample collected at baseline. We were not able to measure conjugated metabolites which may lead to exposure misclassification and limit our ability to compare our results to other studies. While we had detailed anthropometrics at baseline, height and weight during the follow-up period was self-reported, which may have led to random error. However, this was reduced by the annual prospective data collection. Additionally, we had a homogenous population and differences in race and ethnicity could not be examined. This is important since some ethnic groups may be more vulnerable to mycotoxin exposures due to differences in dietary patterns [45], endogenous hormone concentrations [46, 47], and menarcheal timing [48]. In spite of these limitations, this study provides evidence that mycoestrogen exposure may impact girls’ growth and pubertal development. These results demonstrate a strong need for additional research to examine the impact of these chemicals on reproductive development at critical points in the life course.

## Conclusion

Previous experimental evidence shows that mycoestrogens act as endocrine disruptors and their effect may depend on dose, hormonal environment, and critical windows of exposure. However, epidemiological studies examining important windows of exposure during childhood are lacking. Our study is the first one to examine mycoestrogens in relation to age at menarche. We demonstrated that girls with detectable mycoestrogen levels were significantly shorter in stature at menarche compared to girls with undetectable levels. Future studies in humans are extremely important given the evidence of widespread mycoestrogen exposure in human populations.
